# Supplementary material for: Efficient optimization accelerator framework for multi-state spin Ising problems
Source: Nat Commun. 2025 Oct 30;16:9601. doi: 10.1038/s41467-025-64625-2 (PMC12575798; doi:10.1038/s41467-025-64625-2)
Supplement: Supplementary file 1 — Supplementary Information [file 41467_2025_64625_MOESM1_ESM.pdf]

Supplementary Information

Efficient Optimization Accelerator Framework for Multi-state Spin Ising Problems

Chirag Garg[1] ✉, Sayeef Salahuddin[1] ✉

[1]Department of Electrical Engineering and Computer Sciences, University of California, Berkeley, CA 94720, USA.

*** Corresponding author. Email: chirag_garg@berkeley.edu; sayeef@berkeley.edu**

1. Probabilistic Ising Machines

Probabilistic Ising machines follow the principle of the Boltzmann Machine binary neural network [1]. Therefore, the probability distribution corresponding to a given state $p(s) = \frac{1}{Z}e^{-H(s)/T}$ where $Z = \sum_s e^{-H(s)/T}$ is the normalizing/partition function, s represents the states, T denotes temperature coefficient and $H(s)$ is Hamiltonian or energy function to be minimized. Ising Hamiltonian is generally of the form $H(s) = \sum_i \sum_j W_{ij} s_i s_j + \sum_i b_i s_i$, where s_i and s_j represents the state of spins taking binary values {0, 1}, W_{ij} sets the interaction weight between these spins and b_i is bias value for spin s_i. The probability distribution represented by this Ising Hamiltonian is sampled using Gibbs sampling [2] such that the Ising machine stochastically moves toward higher probability states and lower energy. It is enabled by appropriately formulating the update rule, which for Ising Hamiltonian, is given by $P(s_i = 1|s) = sigmoid(-\Delta H_i/T)$ where $sigmoid(x) = 1/(1 + e^{-x})$.

This work particularly employs single flip Gibbs sampling to update the spins as illustrated in Algorithm 2. For each spin update, the algorithm first calculates the change in Hamiltonian (ΔH) due to spin flip and compares $sigmoid(-\Delta H_i/T)$ with a random number from uniform distribution of [0, 1] to update the spin. This comparison predominantly supports the spin flips that cause Hamiltonian to minimize. However, there is still a non-zero probability that it would accept spin updates that cause the Hamiltonian to increase [1,3,4]. It gives rise to minor energy fluctuations in energy exploration data reported in Supplementary Fig. S4.

2. Hyperparameter optimization for graph coloring

In order to make a fair comparison between Ising and vectorized mapping, a grid search-based parameter optimization is performed for the parameters A in Eq. 1 and B in Eq. 2 in the graph coloring Hamiltonian. If the connectivity weighting factor (A) for this constraint is set relatively higher, the Ising machine may frequently violate the one-hot encoding condition. This can lead to the exploration of invalid solution spaces or failure to enforce correct color assignments, resulting in incorrect graph colorings. On the other hand, if one-hot constraint factor (B) is too high, the machine may place too much emphasis on satisfying the one-hot constraint, which can hinder the optimization of the actual coloring objective. As a result, it may become trapped in poor local minima where valid but suboptimal solutions prevail. Supplementary Fig. S1 reports the hyperparameter optimization where we report parameter search for each problems separately by running it for 1000 iteration steps at T = 0.2 and reporting the coloring error (incorrectly colored edges divided by total edges). The optimal settings from Supplementary Fig. S1 are chosen in this work for testing graph coloring problem instances using the probabilistic Ising framework. The simulated bifurcation framework[5] used in this work only accepts the Ising Hamiltonian to minimize. Accordingly, the parameters A in Eq. 1 and B in Eq. 2 are optimized by running the framework for 10000 iteration steps. The optimal values identified in Supplementary Fig. S2 are then used to construct the Hamiltonian, which is subsequently solved using the simulated bifurcation method for the graph coloring problem. For vectorized mapping implementation, only

temperature T effects the results and is chosen equal to 0.2 for all problem instances.

3. Vectorized Mapping Benchmark for Citation Graph Dataset

To demonstrate the scalability of vectorized mapping approach, we benchmark the citation datasets (Cora [6], Citeseer [7], and Pubmed [8]). These problems are often used for graph-based benchmark experiments and have also been used for testing graph-coloring based algorithms [9,10]. Table 2 confirms that the solution accuracy advantage of the proposed vectorized mapping holds for large size graph problems. Supplementary Fig. S5 shows the time-to-solution for these citation graphs. It confirms that the vectorized mapping on GPU takes around one order of magnitude more time compared to the Tabucol heuristics aligning with the scaling trends in Fig. 4c. Moreover, in probabilistic Ising hardware, the overall hardware scaling is primarily influenced by the number of physical nodes required to solve a problem [11,12]. As a result, it follows the scaling behavior of physical nodes with respect to problem size illustrated in Fig. 4a.

4. Traveling Salesman Problem

The Traveling Salesman Problem (TSP) is a classic NP-hard optimization problem that aims to determine the shortest possible route in which a given set of cities is visited exactly once. Owing to its computational complexity and broad applicability in areas such as logistics [13], circuit design[14], and DNA sequencing[15], TSP remains a central focus in combinatorial optimization research. Recently, Ising machines are being explored to tackle this problem [16,17]. To solve N-cities problems, these solvers require N^2 spins modeled via a lattice-like graph representing city number and spin. The Hamiltonian is given as follows:

$$H = A\sum_{k\neq l}\sum_{i} W_{kl}s_{ik}s_{(i+1)l} + B\sum_{i}(1-\sum_{k}s_{ik})^2 + B\sum_{k}(1-\sum_{i}s_{ik})^2 \tag{S1}$$

where A is the connectivity weight factor, B one-hot constraint factor, W_{kl} represents weight defined as distance between city k and l, and $s_{ik} \in \{0,1\}$ is the spin value for visiting k city at i^{th} position in the tour. The first term in the Hamiltonian represents the total weight for given values of spins. However, the second and third terms are to enforce one-hot encoding constraints that penalize assigning multiple cities for visiting at the same i^{th} position and visiting a city multiple times in a tour, respectively. In this work, we use the $burma14$ problem instance from the tsplib dataset [18] and use the first subset of the weight matrix to generate smaller problem instances. The weight matrix is normalized with the maximum distance in it [17]. For the Ising framework, we employ Gibbs Sampling described in Algorithm 2 and choose temperature value 0.02. Further, we exploit the grid-search hyperparameter optimization for parameters A and B as shown in Supplementary Fig. S6. Each problem instance is run for 4000 iteration steps, and optimized for the optimality gap. It is given by the Supplementary Eq. S2, where the optimal solution is calculated using the Lin-Kernighan (LK) heuristics [19].

$$\text{Optimality Gap} = 1 - \frac{\text{tour cost obtained by algorithm}}{\text{optimal tour cost}} \tag{S2}$$

We propose the vectorized mapping framework to tackle the TSP problem, which maps N city problem to $\lceil Nlog_2(N) \rceil$. A spin vector S_i represents the position of the city i in the tour and is defined as $\{s_{i0}, s_{i1}..s_{i(n-1)}\}$ where $n = \lceil log_2(N) \rceil$. Supplementary Algorithm A1 describes the formulation of vectorized mapping Hamiltonian and operator function F. If S_i and S_j are consecutive cities, the Hamiltonian is incentivized to optimize for the tour cost. However, it is penalized when two cities are visited at the same position in the tour guided by the hyperparameter wt. Further, the formulation also restricts S_i to be within its range of N. For the vectorized mapping framework, we employ the Gibbs sampling described in Algorithm 2 and choose the temperature value 0.2. The hyperparameter wt is optimized for the optimality gap, as shown in Supplementary Fig. S7.

To evaluate the broader applicability of the proposed vectorized mapping framework, we conducted additional experiments on the Traveling Salesman Problem (TSP). Both the Ising model and the proposed vectorized mapping were implemented on Nvidia A100 Tensor Core GPU and tested across TSP instances involving up to 14 cities. Each instance was solved 500 times, involving 4000 iteration or update steps per run. As shown in Supplementary Fig. S8, the proposed approach achieves higher success probabilities and shorter time-to-solution compared to the Ising model. Additionally, it consistently produces tours that are closer to those found by the well-established Lin–Kernighan (LK) heuristic, indicating improved convergence toward high-quality solutions (see Supplementary Fig. S9). These results suggest that the vectorized mapping framework is an effective strategy for addressing complex combinatorial optimization problems beyond graph coloring, such as TSP.

Algorithm A1 Vectorized Mapping for Traveling Salesman Problem (F-operator):

$N \leftarrow$ total cities
$H \leftarrow$ Energy Hamiltonian
$wt \leftarrow$ hyperparameter
$n \leftarrow \lceil \log_2(N) \rceil$
$W \leftarrow$ weight matrix for traveling salesman problem
$W_{ij} \leftarrow$ normalized distance between city i and j
$S_i \leftarrow$ position vector for city i in the tour$\{s_{i0}, s_{i1}, \ldots, s_{i(n-1)}\}$
Generate F operator in truth table format for any two city nodes (S_i S_j):
input variables $\leftarrow \{s_{i0}, s_{i1}, \ldots, s_{i(n-1)}, s_{j0}, s_{j1}, \ldots, s_{j(n-1)}\}$
if $|S_i - S_j| == 1$ **then**
    $F \leftarrow 1$ [S_i and S_j cities are next to each other in tour]
    $H \leftarrow H + W_{ij}$
else if $S_i == S_j$ **then**
    $F \leftarrow -1$ [S_i and S_j cities are assigned same position in the tour]
    $H \leftarrow H + wt * (\sum_i W_{ij})$ [penalize the energy hamiltonian]
else if $(S_i, S_j) \notin [0, N-1]$ **then**
    $F \leftarrow -2$ [S_i and S_j cities are assigned outside the time vector range]
    $H \leftarrow H + max(W_{ij})$ [penalize the energy hamiltonian]
else
    $F \leftarrow 0$ [two cites are not next to each other in the tour]
    $H \leftarrow 0$ [does not affect the energy hamiltonian]
end if

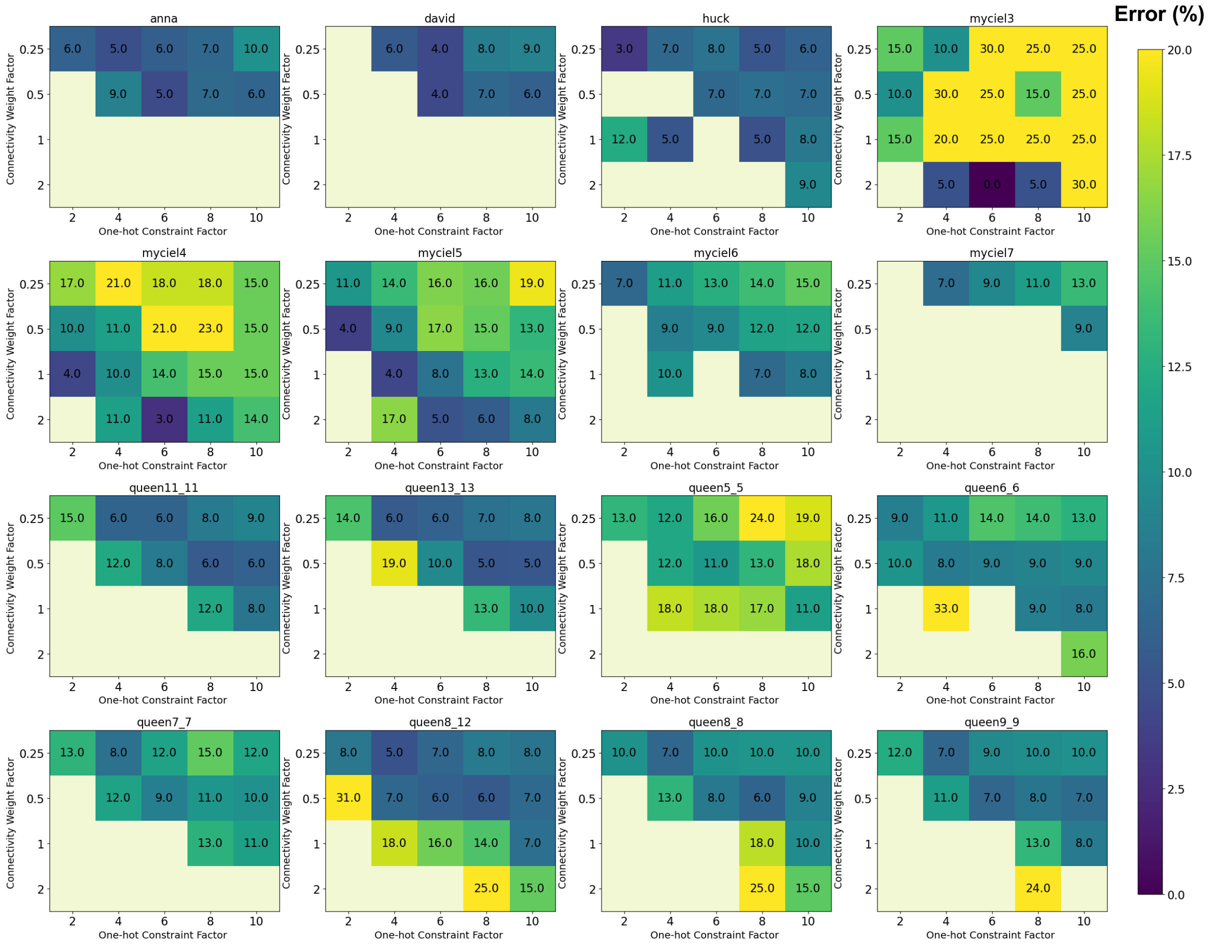

Supplementary Figure S1: Parameter Search across graph coloring problem instances for probabilistic Ising framework. It searches for the optimal value of the connectivity weight factor and one-hot constraint factor to minimize the error. Error is defined as incorrectly colored edges divided by total edges in the problem graph.

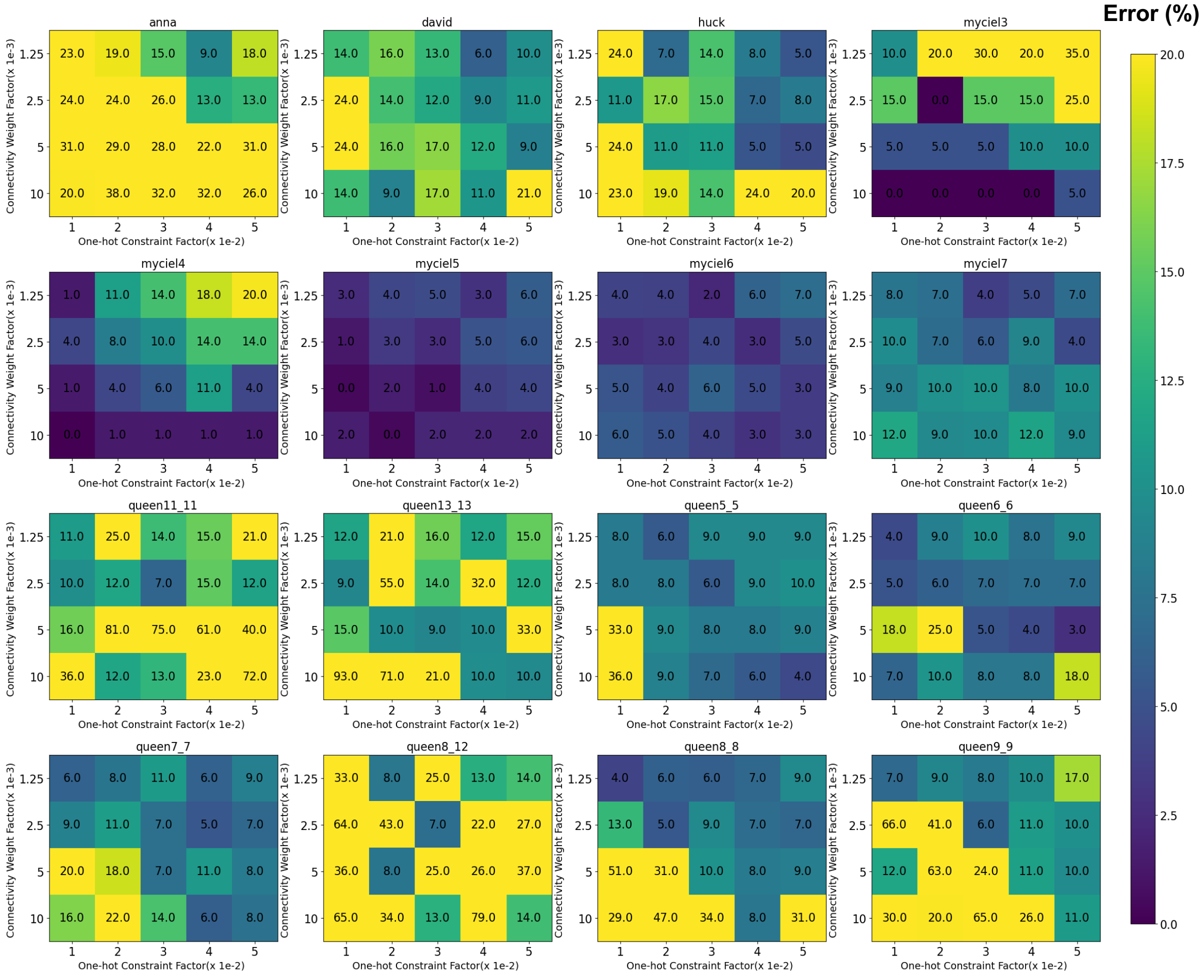

Supplementary Figure S2: Parameter Search across graph coloring problem instances for Simulated Bifurcation framework. It searches for the optimal value of the connectivity weight factor and one-hot constraint factor to minimize the error. Error is defined as incorrectly colored edges divided by total edges in the problem graph.

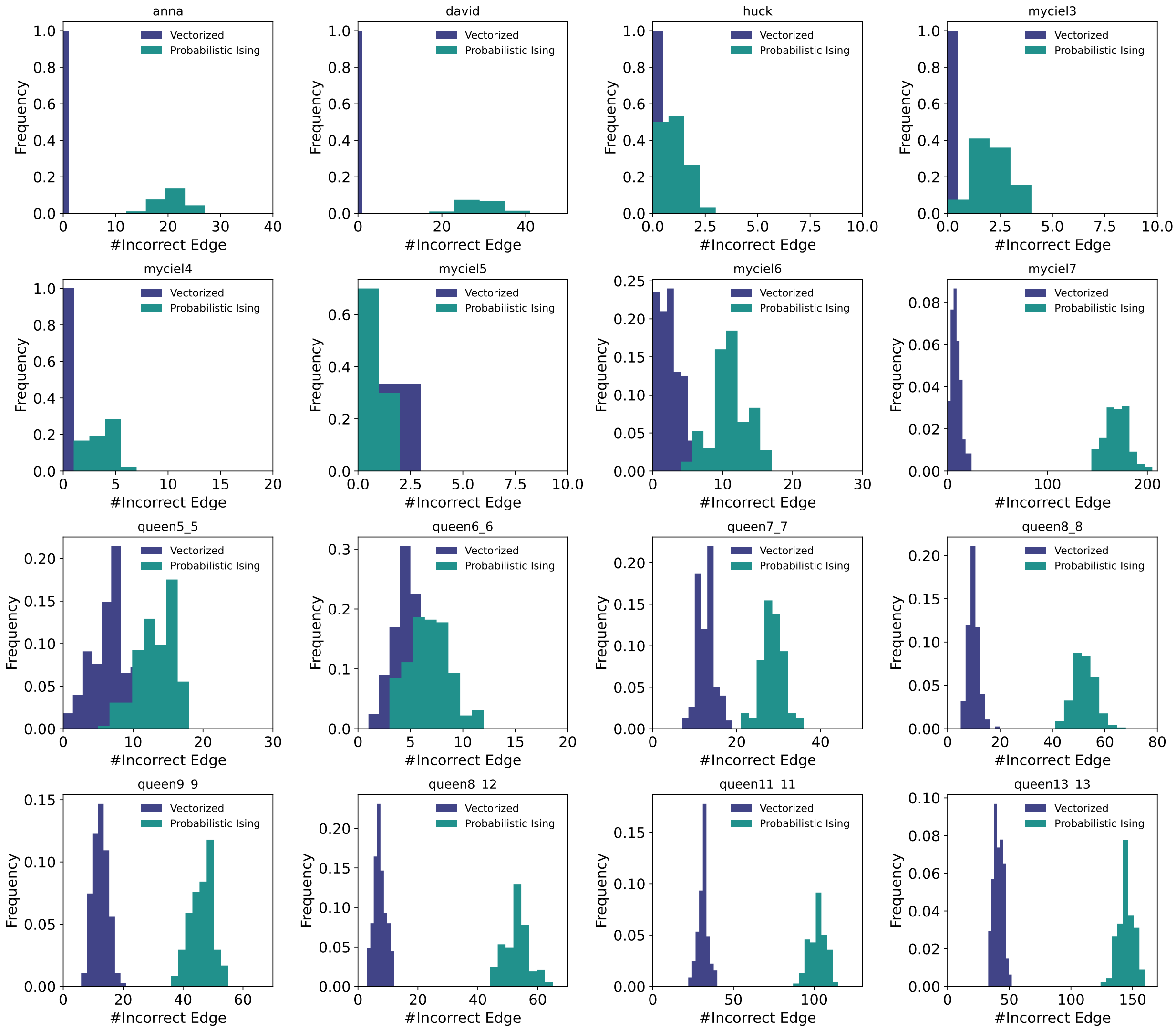

Supplementary Figure S3: Distribution of number of incorrectly colored edges achieved after completing each of 200 parallel runs while solving the graph coloring problem instances [20] on Ising and Vectorized mapping framework.

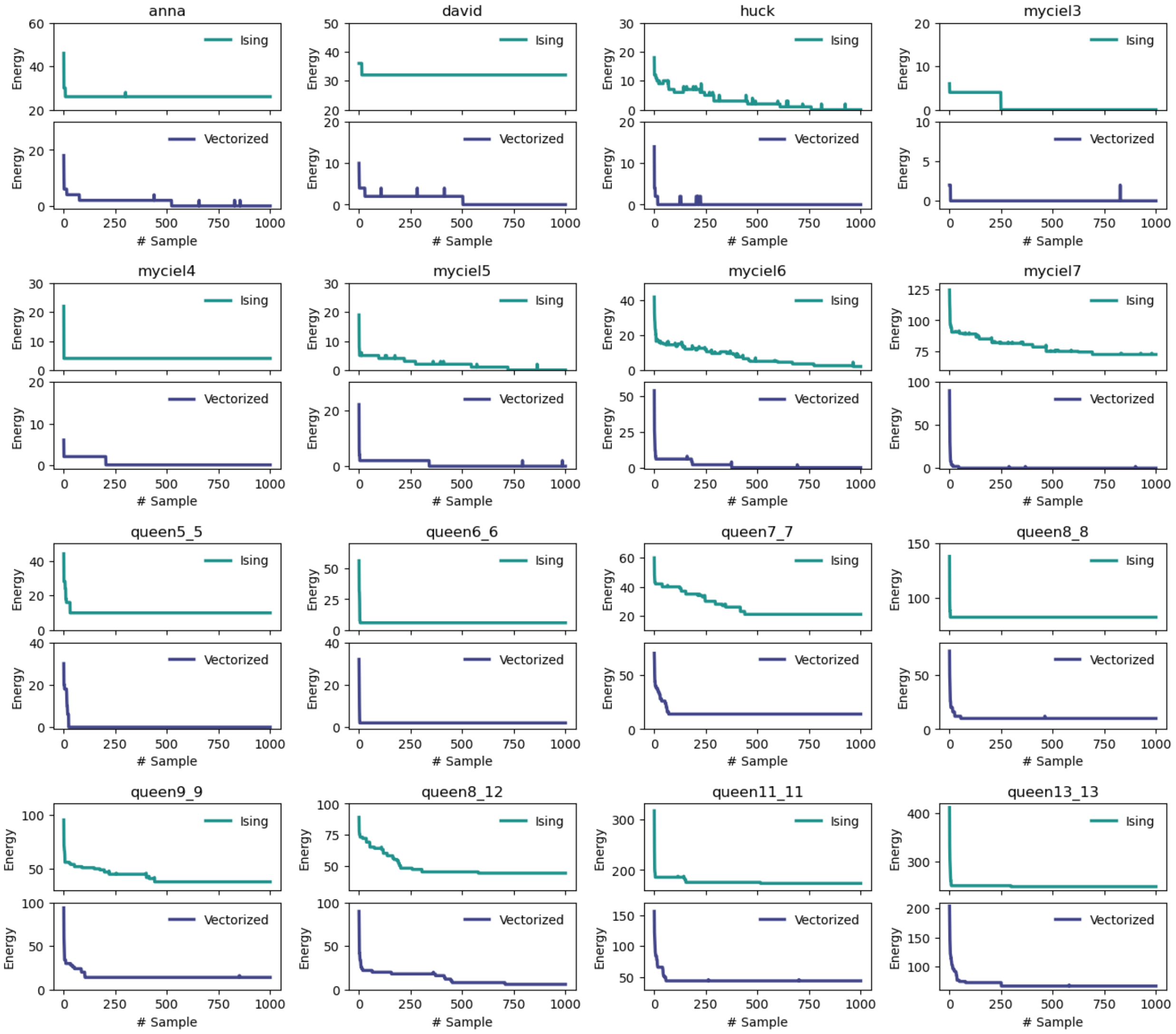

Supplementary Figure S4: Evolution of energy while solving the graph coloring problem instances [20] on Probabilistic Ising and Vectorized mapping framework.

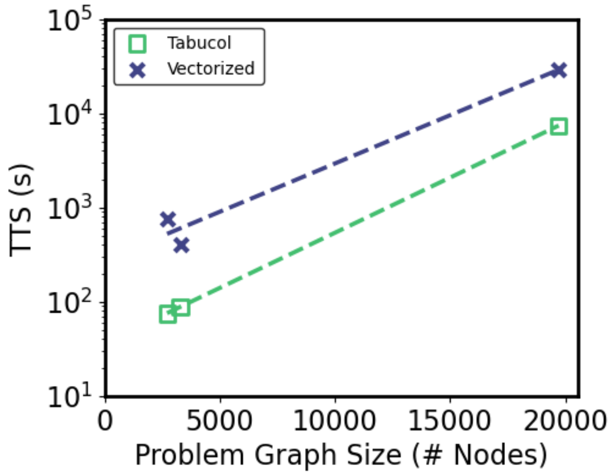

Supplementary Figure S5: Time-to-solution (TTS) for citation graphs benchmarks of vectorized mapping with Tabucol heuristic both implementated on GPU.

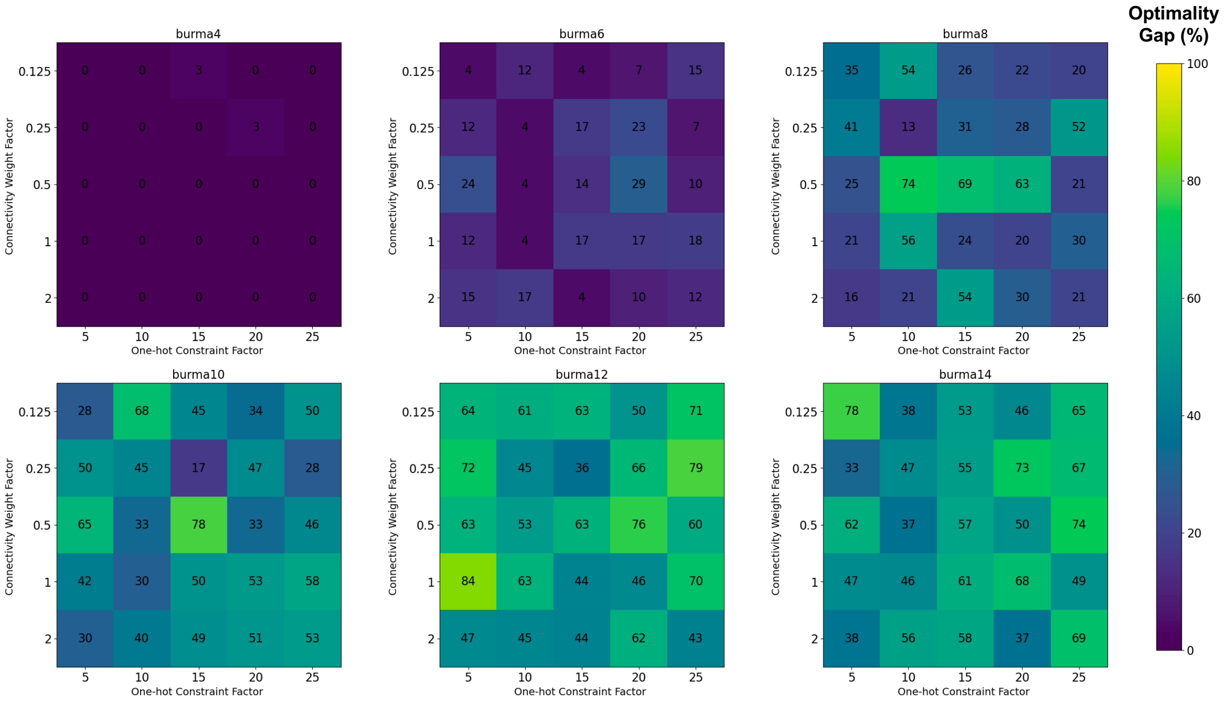

Supplementary Figure S6: Parameter Search across tsp problem instances for Ising framework. It searches for the optimal value of the connectivity weight factor and one-hot constraint factor to minimize the optimality gap. The optimality gap quantifies the relative difference in tour cost obtained by the Ising machines compared with the optimal tour achieved using the Lin-Kernighan Heuristic [19].

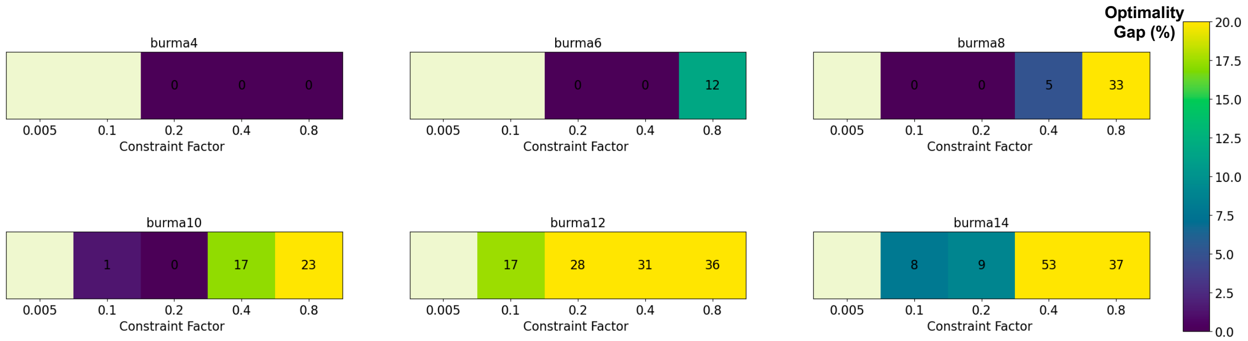

Supplementary Figure S7: Parameter (wt) Search across tsp problem instances for vectorized framework.

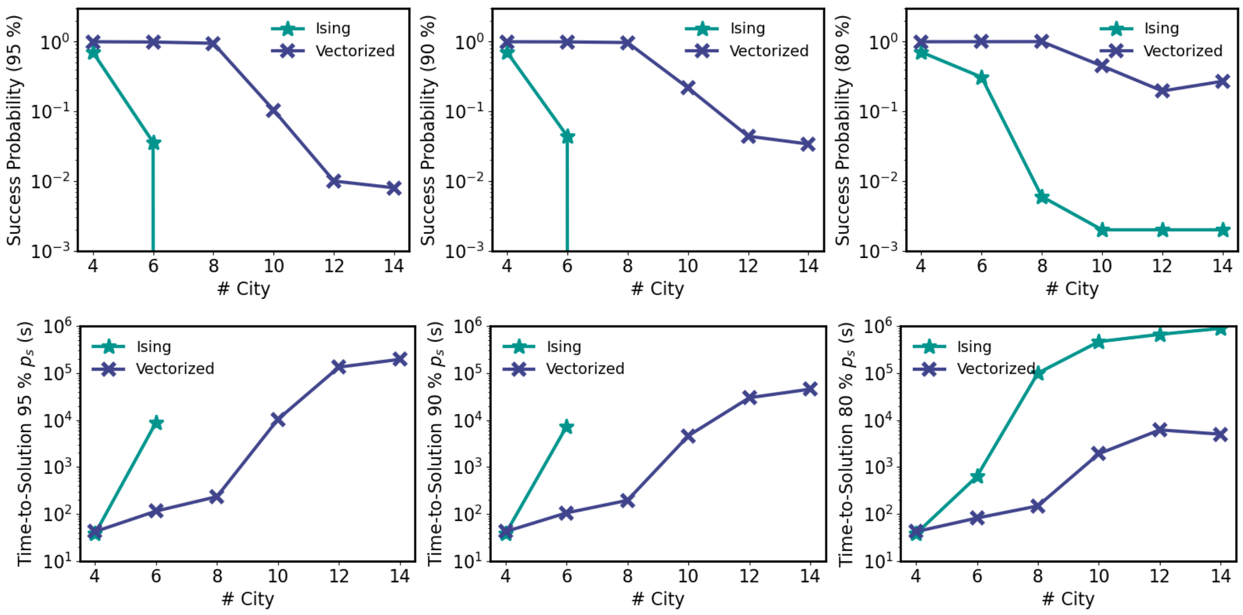

Supplementary Figure S8: Success probability and TTS metric for 99 % success comparing the solution quality and efficiency of Ising and vectorized mapping framework for TSP problem instances upto 14 cities.

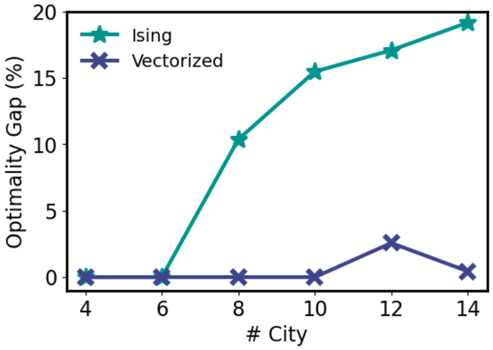

Supplementary Figure S9: Best Optimality gap achieved by Ising and vectorized mapping framework for TSP problem instances upto 14 cities.

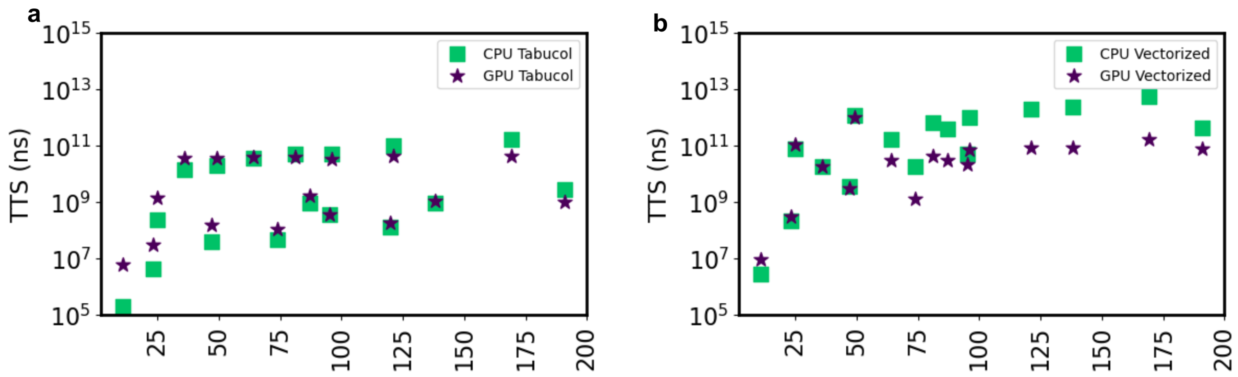

Supplementary Figure S10: Performance comparison between CPU and GPU implementation of Tabucol heuristics and Vectorized framework.

References

1. Ackley, D. H., Hinton, G. E. & Sejnowski, T. J. A learning algorithm for boltzmann machines. *Cognitive Science* **9**, 147–169 (1985).

2. Brémaud, P. Gibbs fields and monte carlo simulation. In *Markov Chains*, 253–322 (Springer New York, New York, NY, 1999).

3. Si, e. a. Energy-efficient superparamagnetic ising machine and its application to traveling salesman problems. *Nature Communications* **15**, 3016 (2024).

4. Borders, W. A. *et al.* Integer factorization using stochastic magnetic tunnel junctions. *Nature* **573**, 390–393 (2019).

5. Ageron, R., Bouquet, T. & Pugliese, L. Simulated bifurcation (sb) algorithm for python. https://github.com/bqth29/simulated-bifurcation-algorithm (2023). Version 1.2.1, Nov. 2023.

6. McCallum, A. K., Nigam, K., Rennie, J. & Seymore, K. Automating the construction of internet portals with machine learning. *Information Retrieval* **3**, 127–163 (2000).

7. Sen, P. *et al.* Collective classification in network data. *AI Magazine* **29**, 93–106 (2008).

8. Namata, G., London, B., Getoor, L. & Huang, B. Query-driven active surveying for collective classification. In *Proceedings of the 10th International Workshop on Mining and Learning with Graphs*, vol. 8, 249–256 (2012).

9. Schuetz, M. J. A., Brubaker, J. K., Zhu, Z. & Katzgraber, H. G. Graph coloring with physics-inspired graph neural networks. *Phys. Rev. Res.* **4**, 043131 (2022).

10. Li, W. *et al.* Rethinking graph neural networks for the graph coloring problem. *ArXiv* (2022). 2208.06975.

11. Li, M.-C. *et al.* 12.2 p-circuits: Neither digital nor analog. In *2025 IEEE International Solid-State Circuits Conference (ISSCC)*, vol. 68, 1–3 (2025).

12. Patel, S. *et al.* PASS: An Asynchronous Probabilistic Processor for Next Generation Intelligence. *ArXiv* (2024). /abs/2409.10325.

13. Lawler, E. L. The traveling salesman problem: A guided tour of combinatorial optimization. *Journal of the Operational Research Society* **37**, 535–536 (1986).

14. Kirkpatrick, S., Gelatt, C. D. & Vecchi, M. P. Optimization by simulated annealing. *Science* **220**, 671–680 (1983). https://www.science.org/doi/pdf/10.1126/science.220.4598.671.

15. Pevzner, P. A., Tang, H. & Waterman, M. S. An eulerian path approach to dna fragment assembly. *Proceedings of the National Academy of Sciences* **98**, 9748–9753 (2001). https://www.pnas.org/doi/pdf/10.1073/pnas.171285098.

16. Whitehead, W., Nelson, Z., Camsari, K. *et al.* Cmos-compatible ising and potts annealing using single-photon avalanche diodes. *Nature Electronics* **6**, 1009–1019 (2023).

17. Dan, A., Shimizu, R., Nishikawa, T., Bian, S. & Sato, T. Clustering approach for solving traveling salesman problems via ising model based solver. In *2020 57th ACM/IEEE Design Automation Conference (DAC)*, 1–6 (2020).

18. Reinelt, G. Tsplib—a traveling salesman problem library. *INFORMS Journal on Computing* **3**, 376–384 (1991).

19. Lin, S. & Kernighan, B. W. An effective heuristic algorithm for the traveling salesman problem. *Operations Research* **21**, 498–516 (1973).

20. Trick, M. COLOR Dataset (2002). Accessed: 2024-09-29.
